# Potential impact of human papilloma virus on survival of basaloid squamous carcinoma of the head and neck

**DOI:** 10.18632/oncotarget.3062

**Published:** 2014-12-18

**Authors:** Christian Jacobi, Isabelle Ayx, Kristin Fritsche, Guido Piontek, Dieter Hoffmann, Gregor Weirich, Andreas Knopf

**Affiliations:** ^1^ Otorhinolaryngology, Ismaningerstr. 22, München, Germany; ^2^ Institute of Virology, Technische Universität and Helmholtz Zentrum München, Trogerstr. 30, 81675 München, Germany; ^3^ Institute of Pathology, Ismaningerstr. 22, München, Germany

**Keywords:** BSCC, basaloid squamous carcinoma, head and neck, survival, HPV

## Abstract

**Objectives:**

Basaloid-squamous-carcinomas (BSCC) have been considered as aggressive variants of common squamous-cell-carcinomas (HNSCC). Recent studies demonstrated a different clinical course depending on the tumour site. The aim of the study is to analyze the histopathologic/clinical features of BSCC/HNSCC resolved by the HPV-status.

**Methods:**

We analysed the histopathologic/clinical features of BSCC (n=59) and HNSCC (n=981), subdivided due to the HPV status. Differences were analysed using Chi square, Fisher exact, and student's t-test. Survival rates were calculated by Kaplan–Meier and log-rank test. Prognostic variables were subsequently evaluated by Cox regression.

**Results:**

Our cohort was congruent with the literature regarding sex, age, metastases, and a predilection in the oropharynx. HNSCC/BSCC did not show a different disease-specific-survival. After UICC matching, univariate analysis revealed a better survival of UICC stage IVa BSCC compared to HNSCC (69% vs. 42%, p=0.022) that was associated with a better response to radio-chemotherapy (p = 0.009). These results referred to the high prevalence of HPV+ (86%) oropharyngeal BSCC. Subgroup analysis demonstrated a better survival of HPV+ oropharyngeal BSCC than HPV-BSCC (p=0.017).

**Conclusion:**

The clinical outcome in BSCC depends on the tumour site and HPV-status. Prospective studies have to evaluate the beneficial application of postoperative radio-chemotherapy in HPV+ BSCC.

## INTRODUCTION

Basaloid squamous cell carcinoma (BSCC) has represented a distinctive subtype of squamous cell carcinoma (SCC) since it was first described by Wain et al. in 1986 [1]. In 2005, the WHO defined BSCC as an aggressive, high-grade variant of SCC composed of both basaloid and squamous components [2]. BSCC demonstrates a predilection for the upper aerodigestive tract, particularly the oropharynx [3, 4]. In the head and neck, BSCC was originally considered a more aggressive and rapidly growing conventional SCC (HNSCC) variant [1, 5]. However, aside from its distinct histopathologic characteristics, the clinical course of the BSCC has been a subject of controversy [6]. Several studies revealed a more aggressive clinical behaviour with low survival rates due to a higher incidence of regional and distant metastases [7, 8]. Other authors did not observe lower survival rates in BSCC and therefore hypothesized a dual behaviour [6]. Recently, multicenter studies that included a larger number of cases possessed the statistical power to show that BSCC seem to follow a completely different clinical course depending on the tumour site. Oropharyngeal BSCC showed a similar or even better prognosis, whereas laryngeal and non-oropharyngeal BSCC demonstrated a poorer survival [9-12]. The molecular mechanisms underlying the clinical phenotype remain unclear. Since human papilloma virus (HPV)-induced HNSCC has been described as a HNSCC-subtype with distinct epidemiological, clinical, and molecular characteristics, resulting in a more favourable prognosis, the impact of HPV in BSCC was also examined [13, 14]. Some authors have recently suggested a favourable prognosis in HPV-induced BSCC. But these studies analysed small numbers of cases and survival data were not matched with HNSCC so far [15, 16]. Studies that comprise a sufficient sample-size to stratify the cohort by the tumour site, stage, and HPV status are lacking. The purpose of this study is to analyze the histopathologic and clinical features of a large BSCC cohort, which has been resolved by HPV status and matched with conventional HNSCC data. The study mediates between the database-assisted investigations that show efficiently the site-dependency of BSCC behaviour and the small mono-institutional studies representing inconsistent data.

## RESULTS

### Epidemiologic and clinic-pathologic features

Patients and tumour characteristics are shown in table 1. The subgroup of oropharyngeal BSCC was analysed additionally. In both groups, the majority of patients were males (p = 0.172). In the overall cohort BSCC, patients tended to be older at the initial presentation (BSCC: 62 years vs. HNSCC: 59 years, p = 0.093), with a significant difference in oropharyngeal BSCC (64 years vs. oropharyngeal HNSCC: 59 years, p = 0.002). Smoking and drinking behaviour was equally existent in both groups (smoking status p = 0.609, alcohol status p = 0.320). BSCC showed a striking predilection for the oropharynx (p < 0.001). HNSCC demonstrated a balanced site distribution (Tab. 1). BSCC were mostly staged as T2 tumours (all locations: 41%, oropharyngeal BSCC: 51%). HNSCC demonstrated a balanced t-stage distribution (Tab.1). In contrast to HNSCC, the rate of lymph node metastases was significantly increased in BSCC (BSCC/HNSCC: 88%/59%, p < 0.001; oropharyngeal BSCC/ oropharyngeal HNSCC: 93%/72%, p = 0.002) and demonstrated stage N2(a-c) metastases with a pronounced occurrence in BSCC/oropharyngeal BSCC. The laryngeal and hypopharyngeal subpopulation also showed a higher rate of lymph node involvement (BSCC: 90%; HNSCC: 56%, p = 0.028). The high prevalence of an advanced lymph node involvement in BSCC was highly significant (all locations: p < 0.001; oropharynx: p = 0.003). Subgroup analysis revealed lymph node metastases in 85% of T1-2 staged BSCC, while HNSCC showed a lymph node involvement in 48% of T1-2 tumours (p < 0.001). As the incidence of distant metastasis were similar in both groups (5% BSCC vs. 4% HNSCC, p = 0.384), the N status mostly determined the advanced UICC stage (BSCC: 70% UICC 4a; HNSCC: 48% UICC 4a). Concordant with the underlying N status the distribution of UICC stages differed significantly between BSCC and HNSCC (p = 0.002). In the BSCC group 10 patients (23%) were R+/Rx, 107 patients (17%) in the HNSCC group, respectively (p = 0.530) (Tab.1).

**Table 1:** Comparison of the epidemiologic and tumour characteristics of the BSCC/HNSCC cohort

Demographic and tumor characteristics	All locations		Oropharynx		p-value		HPV+	
	BSCC	HNSCC	BSCC	HNSCC	All loc.	Oroph.	BSCC
n	59	981	41	370			
**Age (years)**							
Median [25%; 75%]	62[54; 70]	60[52; 66]	65[58;71]	59[53; 66]			
Mean ± SD	62±9,4	59±10,4	64±8,1	59±9,5	0.093	0.02	
**Sex,** n (%)							
Male	43(73)	787(80)	31(76)	286(77)	0.172	0.807	
Female	16(27)	194(20)	10(24)	84(23)			
**Risk factors**							
Nicotine abuse	36 (77)	304 (80)	25 (81)	107 (77)	0.609	0.658	
Alcohol abuse	30 (64)	270 (71)	19 (61)	100 (71)	0.320	0.242	
**Location**							
Sinunasal	2(3)	30(3)			<0.001		1 (3)
Nasopharynx	1(2)	19(2)					0
Oropharynx	41(70)	370(38)					31 (86)
Hypopharynx	6(10)	211(22)					1 (3)
Larynx	6(10)	202(21)					2 (6)
Oral cavity	3(5)	149(15)					1 (3)
**Second primary tumors**	12(20)	270(28)	9(22)	107(29)	0.228	0.347	
**T stage**							
Tx	0	3	0	2	0.170	0.055	
T1	10(17)	260(27)	7(17)	91(25)			
T2	24(41)	287(29)	21(51)	116(32)			
T3	10(17)	208(21)	4(10)	78(21)			
T4	15(24)	223(23)	9(22)	83(23)			
**N stage**							
N0	7(12)	403(41)	3(7)	104(28)	< 0.001	0.003	
N1	9(15)	120(12)	6(15)	58(16)			
N2a-c	43(73)	426(44)	32(78)	191(51)			
N3	0	32(3)	0	17(5)			
**M stage**							
M0	31(53)	603(62)	21(51)	233(63)	0.384	0.206	
M1	3(5)	36(4)	1(2)	16(4)			
Mx	25(42)	342(35)	19(46)	121(33)			
**Grading**							
Gx		24(2)		6(2)			
G1		44(5)		10(3)			
G2		503(51)		184(50)			
G3	59(100)	397(41)		167(45)			
G4		13(1)	41(100)	3(1)			
**R stage**							
R0	33 (77)	523 (83)	23 (72)	181 (79)	0.530	0.380	
R1	6 (14)	48 (8)	6 (19)	22 (10)			
R2	1 (2)	14 (2)	0	5 (2)			
Rx	3 (7)	45 (7)	3 (9)	20 (9)			
**UICC**							
Stage 1	1(2)	157(16)	0	36(10)	0.002	0.069	
Stage 2	4(7)	128(13)	2(5)	43(12)			
Stage 3	8(14)	165(17)	6(15)	68(19)			
Stage 4a	41(70)	468(48)	30(73)	188(51)			
Stage 4b	2(3)	28(3)	2(5)	17(5)			
Stage 4c	3(5)	32(3)	1(2)	16(4)			
**Treatment**							
OP only	0	185(19)	0	47(13)	<0.001	<0.001	
OP + RTX	19(32)	269(27)	12(29)	97(26)			
OP + RCTX	24(41)	179(18)	20(49)	85(23)			
Prim. RCTX	14(24)	315(32)	7(17)	135(37)			
Prim. RTX	2(3)	33(3)	2(5)	6(2)			

### Treatment modalities

Treatment modalities (OP ± R(C)TX or primary R(C)TX) differed within both groups (p < 0.001). BSCC patients underwent more often an adjuvant RCTX (BSCC/oropharyngeal BSCC: 41%/49%; HNSCC/oropharyngeal HNSCC: 18%/23%). Beside the higher frequency of postoperative positive margins (n = 9) within the BSCC group, other risk factors such as extra-capsular spread (n = 6), angio-invasion (n = 2), lymphangiosis carcinomatosa (n = 5) or a combination (n = 2) led to the prevalent decision towards combined modality adjuvant treatment. Surgery without an adjuvant treatment was done in 19% of the HNSCC and never in the BSCC group. In BSCC 50 patients (85%) completed therapeutic procedures, 874 patients (89%) in the HNSCC group.

### Survival analysis

**Figure 1:**
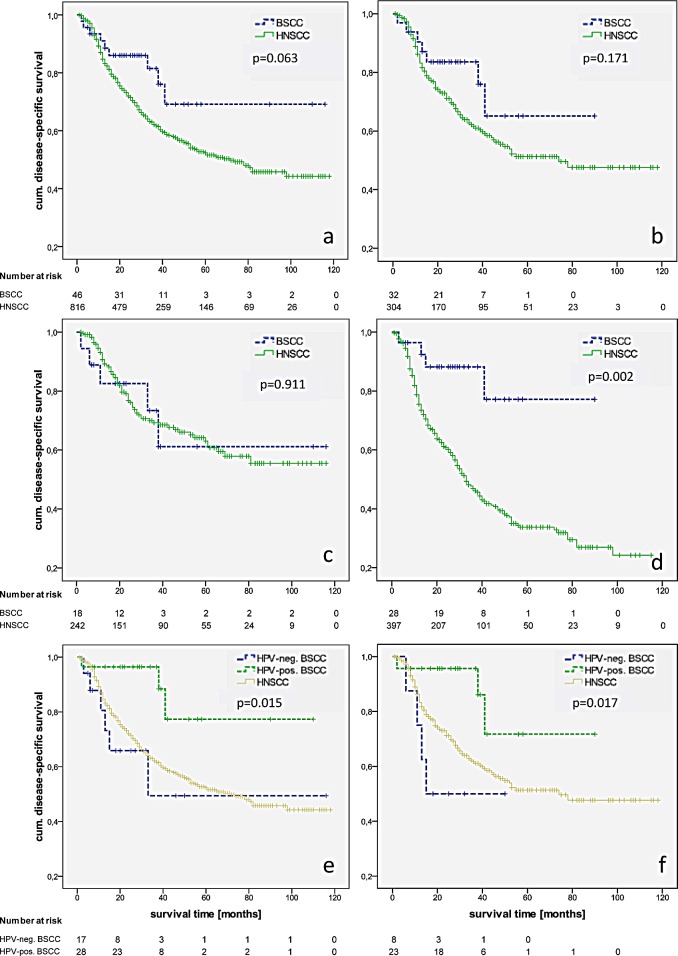
Kaplan-Meier estimates of disease-specific survival in BSCC/HNSCC in all locations (a) and the oropharynx (b), in all locations with OP and RTX (c) and OP+RCTX/prim RCTX (d), and HPV-dependent disease-specific survival DSS of BSCC in all locations (e) and the oropharyngeal subgroup (f).

Recurrence rates and the 5-year RFI were similar in BSCC and HNSCC as well as the oropharyngeal subgroups (27%/30% and 58%/61%, p = 0.866). After a mean follow-up of 32 months in the BSCC and 33 months in the HNSCC group, there were no significant differences in OS between BSCC and HNSCC (all locations: p = 0.190; oropharynx: p = 0.213) and a trend to a better DSS for BSCC (all locations: 69% vs. 52%, p = 0.063; oropharynx: 65% vs. 51%, p = 0.171) (Fig. 1a, b). Matching the groups according to the UICC stage, univariate analysis revealed a significant better survival of UICC stage IVa BSCC compared to the HNSCC counterparts (69% vs. 42%, p = 0.022). While patients who underwent an adjuvant RTX demonstrated similar survival rates, BSCC patients with an adjuvant RCTX or primary RCTX showed a significant favourable outcome (p = 0.009) (Fig. 1c, d). Multivariable DSS analysis was possible for a total of 862 patients. In the entire group, BSCC was determined to have a significant better outcome after adjustment for other independent risk factors like T-, N-, and M-classification (HR [95%CI]: 0.476 [0.244-0.927], p = 0.029) (table 2).

**Table 2:** Multivariable DSS analysis of 862 BSCC/HNSCC patients after adjustment for T-, N- and M-classification

Variable	Univariate analysis DSS *(p)*	Multivariate analysis DSS *(HR)*		
		HR	95%-CI	p
**All locations**				
HNSCC vs BSCC	0.063	0.476	0.244-0.927	0.029
T-stage: T1/2 vs. T3/4	<0.001	2.411	1.895-3.068	<0.001
N-stage: N0 vs. N+	<0.001	1.842	1.418-2.394	<0.001
M-stage: M0 vs. M+	<0.001	2520	1.564-4.058	<0.001

### p16, HPV16/18, and survival

Sixty-two per cent of the BSCC (n = 58) were stained p16-positive. We identified a significant p16 predilection in the oropharynx (86%). Seventy-two per cent of the non-oropharynx BSCC was p16-negative (p < 0.001). p16 positive samples were verified by HPV 16/18 DNA analysis, resulting in a HPV 16 positivity for all cases and one case with a HPV 16 and 18 positivity. There was no difference between p16 negative and positive BSCC regarding sex, age, and UICC stage. Smaller T-stages (T1-2) with loco-regional metastases were frequently observed in p16-positive BSCC (p = 0.017). The 5-year survival rate for all head and neck tumour sites was 79.3% for p16 positive BSCC and 42.0% for p16 negative BSCC (p = 0.015). Subgroup analysis of oropharyngeal BSCC revealed a 5-year survival rate of 71.7% for p16 positive individuals and 50.0% of p16 negative patients (p = 0.017). The 5-year survival rate in HNSCC was 51.3%. Univariate analysis did not indicate age, sex, stage, and localisation as significant prognostic factors. Unadjusted hazard ratios for p16-negative vs. p16-positive BSCC were assessed in the Cox regression model, revealing p16-status as an influential factor on DSS (all locations HR 0.210 [95%CI: 0.052-0.853], p = 0.029; oropharynx HR 0.185 [95%CI: 0.040-0.858], p = 0.031) (Fig. 1 e, f). Furthermore, patients with p16-positive BSCC had a 64% reduction in the risk of death (OS) referred to p16-negative ones.

## DISCUSSION

BSCC was first described 30 years ago, but controversies about the molecular behaviour and patients' prognosis continue. Our BSCC cohort was congruent with the literature regarding sex, age, and a predilection in the oropharynx (70%). Fritsch et al. stated that the increasing incidence of oropharyngeal BSCC seems to reflect the recent epidemic of HPV related oropharyngeal HNSCC and reinforced the hypothesis by sub-analyzing the distribution of the primary site by year. Fritsch et al. showed an increasing proportion of oropharyngeal BSCC from 2000 to 2008 [3]. The high incidence HPV-positive oropharyngeal BSCC presented in our cohort confirms these findings. Several studies point out that BSCC occur in advanced stages, citing wide-ranged frequencies of lymph node positive cases from 25%-83% (Supp. 1) [1, 5, 8-11, 17-21]. We can specify these findings with an extraordinarily high incidence of lymph node positivity in BSCC compared to HNSCC. We detected BSCC in smaller T stages than HNSCC, which validates the results of Thariat et al. and Fritsch et al [3, 4]. Our study showed that especially small tumour sizes in BSCC are more frequently accompanied with positive N-stages than HNSCC-tumours (p<0.001). The BSCCs' propensity for early lymph node metastases has been described by several authors, but the underlying biology remained unclear. Analogous studies on HNSCC showed the association between HPV and early lymph node spread [4, 22]. Simultaneously, the high rates of lymph node involvement in BSCC compared to HNSCC appear not only site-specific in oropharyngeal BSCC but also in laryngeal and hypopharyngeal sites, as revealed in our study. As these facts are congruent with the results of other studies, it can be assumed that there are concomitant molecular mechanisms besides the HPV infection [11, 12]. The rates of distant metastases are analogically discussed. While prior studies state distant metastases in BSCC of 10%-50%, recently published site-specific multicenter data could present lower rates of 5% in oropharyngeal BSCC to 12% in laryngeal lesions that are comparable to our findings [9-11, 17-20, 23]. Neither our results nor those of Fritsch et al. could identify different metastases rates in oropharyngeal HNSCC when compared with oropharyngeal BSCC. In contrast, laryngeal and oral BSCC seem to develop more metastases, which may contribute to the worse clinical course of those tumours [9, 11]. Another reason for the controversial data could be traced back to an inconsistent definitional use of the term ‘distant metastasis’. In our literature review, we determine whether the respective authors refer to the M-status at initial presentation or during the follow-up (Supp. 1). Although our BSCC patients had significantly higher rates of lymph node involvement compared to HNSCC, we could not show a correlation with higher rates of recurrences and mortality. Regarding the RFI, there were no differences between BSCC and HNSCC, which is concordant to the current data [4, 7, 24]. In our study, the OS/DSS for BSCC followed a similar to better clinical course in BSCC than in HNSCC. After matching the patients by UICC stage, BSCC were associated with an even better outcome (p=0.002). In order to set our survival data into context with prior studies that led to the hypothesis of the BSCCs' dual behaviour, we assessed 24 studies dealing with the clinical outcome (Supp. 2). A total of 10 studies stated a worse (two studies with significant results) and 10 a similar BSCC outcome, whereas two recent studies revealed significant better survival for site-specific oropharyngeal BSCC. Only two case-control studies have been performed so far. Soriano et al. suggest a significantly worse outcome for BSCC cohort consisting of 50% hypopharyngeal tumours [7]. In contrast, Thariat et al., who investigated 51 patients (85% oropharyngeal BSCC) with lymph node involvement, could not find any differences in patients' prognosis or the rates of distant metastases compared to HNSCC [4]. These data are restricted as the primary treatment modality was radiotherapy and surgery was only performed for neck dissection [4]. In contrast to these mono-institutional studies performed before 2013, recent publications are premised on huge sample-sized multi-centric databases and all describe a significant favourable outcome for oropharyngeal BSCC. Fritsch et al. showed site-specific differences that clearly contradict the common characterization of the BSCC as a uniformly aggressive neoplasm [3]. Besides the laryngeal lesions, Fritsch et al. also revealed that BSCC of other sites carry a similar or better prognosis [3]. The potential impact of a HPV related carcinogenesis in oropharyngeal BSCC was analysed by Chernock et al. and Begum et al., who detected a relationship between HPV and BSCC in 75/76% of the oropharyngeal lesions and 0/6% in non-oropharyngeal sites, resulting in a significantly better survival in HPV positive tumours [15, 16]. Based on the largest BSCC cohort resolving the HPV status so far, we could show an even higher prevalence of HPV positive oropharyngeal BSCC (86%) associated with smaller tumour sizes and a high incidence of lymph node spread. These findings indicate a higher rate of HPV infection in oropharyngeal BSCC compared to oropharyngeal HNSCC, whose HPV incidence typically ranges from 32%-36% [25, 26]. We could reveal that HPV positive BSCC are strongly associated with a better prognosis. Due to the predominance of HPV infection in the oropharynx, studies including high proportions of oropharyngeal BSCC subsequently contribute to the inconstancies in clinical outcome among the literature [4, 10, 15, 17, 27, 28]. There is scant information about the specific efficacy of treatment modalities on BSCC. We could statistically figure out that BSCC benefit from adjuvant radio-chemotherapy in a dimension exceeding that of HNSCC (p=0.009). Stratifying the outcomes only for adjuvant radiotherapy, the mortality was equal. Certainly, due to limitations of our retrospective study, these results can merely contribute to the upraising discussion of radio-(chemo) sensibility of BSCC. On the basis of a case-control study, Thariat et al. demonstrated the given radio-sensibility and also described similar outcome rates for BSCC and HNSCC. However, the authors did not control for a potential effect of adjuvant chemotherapy, which was also implemented in 45% of the cases [4]. Since prior studies did not stratify their findings for treatment modalities including chemotherapy, comparable data are lacking. We hypothesize an increased radio-(chemo) sensibility in BSCC, although we could not detect a dependency either by the basaloid histology or by HPV positivity. It must be considered that the paradigm of BSCC as a highly aggressive entity can lead to a more radical clinical treatment scheme and could therefore bias comparative survival data. Prospective studies must focus on treatment modalities in dependency of the HPV status and tumour site to answer how far BSCC benefit from postoperative radio-chemotherapy. BSCC of the head and neck should be separated in HPV positive oropharyngeal BSCC, HPV negative oropharyngeal BSCC and Non-oropharyngeal BSCC, since BSCC cannot be regarded as a uniformly entity any longer.

## CONCLUSION

The facts of both a high occurrence of lymph node metastases (despite small tumour sizes) that are not associated with a poorer prognosis and the coincident finding of a tremendous prevalence of HPV positive oropharyngeal BSCC underline the impact of HPV in the carcinogenesis of BSCC. The clinical outcome is mainly dependent on the tumour site as oropharyngeal BSCC follow an even better course than oropharyngeal HNSCC that can be traced back on the HPV status.

## MATERIAL & METHODS

### Patient selection

The study included 59 patients with BSCC and 981 with HNSCC that were diagnosed in the ENT department of the University Hospital Rechts der Isar, Munich, during a period of 10 years (01.01.2001–01.09.2011). The diagnosis of BSCC was based on the morphologic criteria defined by Wain [1]. BSCC tumour samples were histologically reviewed by at least two experienced pathologists. Dysplasia, carcinoma in situ, and other histologic subtypes such as adenocarcinoma were excluded from the study. Clinical parameters and survival data were retrospectively collected: age, sex, alcohol and nicotine abuse, second primary tumours, TNM-Staging, grading, treatment modalities, recurrence, and death/loss to follow-up. Patients with lacking data, incomplete staging, and refused/not finished surgical and/or conservative treatment (radio-/chemotherapy) treatment were excluded from survival analysis. The median and mean follow-up time were 24 [11; 45] and 32 months. Paraffin-embedded tumour (FFPE) samples from 58 BSCC-patients were available for p16-immunhistochemisty and HPV16/18-genotyping.

### Statistical analysis

Differences between the BSCC and HNSCC groups were analysed using the Chi square test and Fisher exact test for categorical, and the unpaired student's t-test for continuous variables. As main endpoints the overall survival (OS), disease-specific survival (DSS) and recurrence-free interval (RFI) were assessed measuring the time from treatment to death of any cause, tumour-related death and locoregional recurrence, and/or distant metastasis. Survival rates and curves were calculated and illustrated by the Kaplan–Meier method and further analysed by the log-rank test for univariate analysis. Variables that revealed prognostic or effect modifying potential on the outcome as suggested by univariate analysis were subsequently evaluated by the proportional Cox regression for multivariate analysis. p-values <0.05 were considered statistically significant. Statistical analysis was done using SPSS (SPSS Inc., Chicago, IL).

### p16-immunohistochemistry

FFPE tumour sections (3 μm) were p16 (Ventana, AZ, USA) stained and visualized with the Bond Polymer Refine Detection kit (Leica, Nussloch, Germany). Tissue samples with known p16 expression were used as positive controls. To describe the expression levels we used a scoring system classifying the staining intensity (0 = no staining, 1 = low, 2 = moderate, 3 = strong staining intensity) and the relative proportion of stained cells (0, 1 = <10%, 2 = 10%–39%, 3 = 40%–69%, 4 = >70 of the tumour cells). A cumulative score (range 0–7 points) was assessed by adding both scores. A positive staining was defined by a cumulative score equal or greater than 3.

### HPV16-in-situ hybridization

Small tissue samples that were not suitable for DNA extraction and molecular HPV-16/18 analysis underwent HPV in situ hybridization for high-risk subtypes 16, 18, 31, 33, and 51 (Leica) on a Ventana Benchmark XT automated stainer (Ventana). Tissue samples with an intense nuclear staining of at least 10% tumour cells were defined as positive.

### DNA extraction from FFPE

DNA was isolated by mixing five 10 μm FFPE sections with 1 ml Xylol and adding 250 μl 70% ethanol. After vortexing, the tubes were centrifuged (19330 g, 5 min) and the supernatant was carefully decanted. A vacuum centrifuge was used to dry the probes for 60 min at 45°C. The dried pellet was re-suspended in 600 μl Proteinase K buffer (Qiagen, Hilden, Germany) + 25% Tween (Carl Roth GmbH & Co, Karlsruhe, Germany). Next 50 μl Proteinase K (Qiagen) was added and incubated over night at 55°C. Enzyme activity was inhibited at 95°C for 10 min.

### HPV16/18-PCR

HPV16/18-DNA was detected by PCR amplification. For high-risk genotypes, we used the Abbott Realtime HR HPV assay running on the m2000sp instrument (Abbott, Wiesbaden, Germany). The analytical sensitivity depends on the subtype: 500 copies per assay for 16, 18, 35, 39, 45, 51, 59, 66, 68; 2000 copies per assay for 31, 33, 52, 56, and 5000 copies per assay for 58, according to the manufacturer. Three forward and two reverse primers target a conserved L1 region. The endogenous human beta globin gene controls for sufficient cell number in the sample, nucleic acid extraction, and amplification efficiency. Tissue samples that were positive for p16 and HPV16/18 DNA were considered HPV positive.

### Literature review

A selective literature review in PubMed Central Web addressing BSCC of the head and neck was performed. ‘Basaloid squamous carcinoma’, ‘BSCC’, ‘head and neck’, ‘upper aerodigestive tract’, ‘epidemiology’, ‘survival’, and ‘outcome’ were used as key words. We included all available articles considering more than seven patients and reviewed them for clinic-pathological and survival data (Supplements 1, 2).

## SUPPLEMENTARY MATERIAL


