# Supplementary material for: Potential impact of human papilloma virus on survival of basaloid squamous carcinoma of the head and neck
Source: Oncotarget. 2014 Dec 18;6(5):3462–70. doi: 10.18632/oncotarget.3062 (PMC4413667; doi:10.18632/oncotarget.3062)
Supplement: Supplementary file 1 [file oncotarget-06-3462-s001.pdf]

Potential impact of human papilloma virus on survival of basaloid squamous carcinoma of the head and neck

Supplementary Material

Author Year Type	n BSCC	n conventional SCC	Men in %	Age Mean	Localisation	N+ in %	Diff. (N+)	M initial/ follow-up	Diff. (M+)	Sec. CA in %
Fritsch 2013 CO	650	7282 P-SCC, 8819 M-/W-SCC	87	60	100% Oropharynx	74	worse (s)	5/-	Similiar	NA
Linton 2014 CO	642	33554 P-SCC	80	62 Med	76% Oropharynx 12% Oral cavity 12% Larynx/ Hypopharynx	82 37 51	Worse (ns) similiar worse (s)	NS	NA	NA
Fritsch 2013 CO	145	3815 P-SCC, 13282 M-/W-SCC	77	64	100% Larynx	49	worse (s)	12/-	Worse (S)	NA
Fritsch 2013 CO	92	2340 P-SCC, 11026 M-/W-SCC	74	67	100% Oral cavity	40	similiar	7/-	Worse (S)	NA
Soriano 2008 CC	62	6 P-SCC, 16 M-SCC, 40 W-SCC	95	61	50% Hypopharynx, 31% Oropharynx/ Oral cavity	52	matched	-/45	Worse (NS)	NA
Thariat 2008 CC	51	102 P-SCC, 102 M-/W-SCC	86	57 Med	85% Oropharynx	100	matched	0/29	matched	8
Winzenburg 1998 CO	26	23 P-SCC	NA	NA	NA	61	NA	10/52	Worse (NS)	NA
De Sampio 2004 CO	17	27 P-SCC, 27 M-SCC	88	59	1005 Oral cavity	70	NA	NA	NA	18
Yu 2008 CO	15	15 SCC (NA)	100	60 Med	66% Oral cavity, 27 % Sinonasal	67	worse (ns)	13/-	similiar	13
Erdamar 2000 CO	10	44 W-SCC	100	61	100% Larynx	40	similiar	10/-	Similiar	NA
Luna 1990 CO	9	47 SCC (NA)	78	61	65% Hypopharynx, 44% Oropharynx	83	NA	10/-	NA	NA
Marioni 2008 CO	9	9 SCC (NA)	100	66 Med	100% Larynx	44	similiar	0/-	similiar	0
Coppola 1993 CO	8	41 P-/M-SCC, 22 W-SCC	63	52	100% Oral cavity	75	similiar	0/38	similiar	NA
Begum 2008 CS	53	-	85	60 Med	40% Oropharynx, 26% Hypopharynx	NA	NA	NA	-	NA
Ereno 2008 CS	40	-	88	60	55% Larynx/ Hypopharynx	63	NA	6/-	-	18
Banks 1992 CS	40	-	88	62 Med	50% Oropharynx, 20% Hypopharynx	68	NA	-/38	-	5
Chernock 2009 CS	28	-	64	NA	43% Oropharynx, 57% Hypopharynx/ Larynx	NA	NA	NA	-	NA
paulino 2000 CS	20	-	70	62	25% Oropharynx, 25 %Larynx	25	NA	5/-	-	0
Choussy 2011 CS	18	-	78	61	38% Hypopharynx 38%Larynx	50	NA	6/39	-	NA
Friedrich 2010 CS	17	-	59	62	17% Oropharyx, 17% Oral cavity	NA	NA	NA	-	NA
Ferlito 1997 CS	15	-	87	63	60% Larynx, 40% Hypopharynx	60	NA	-/60	-	NA
Larner 1993 CS	15	-	87	60	38% Oropharynx, 13% Larynx	73	NA	-/53	-	NA
Wieneke 1999 CS	14	-	50	67 Med	100% Nasal Cavity/Sinonasal	NA	NA	-/36	-	NA
Klijanienko 1993 CS	12	-	92	56	33% Hypopharynx, 33% Oropharynx	75	NA	NA	-	NA

Supplement 1: Literature review for epidemiologic and clinicopathoIic characteristics. CO = comparative study; CC = case-control study; CS = case series; p- (poorly-), m- (moderate-), w- (well-) SCC, Med=median, NA=not assessed, (s)=significant, (ns)=not significant

Author	n BSCC	Localisation	Prim. Treatment	Diff. recurr	Survival outcome	HPV	HPV Impact
**Fritsch 2013 CM**	650	100% Oropharynx	OP/±RTX	NA	better (s)	NA	NA
**Linton 2014 CM**	642	76% Oropharynx<br>12% Oral cavity<br>12% Larynx/ Hypopharynx	OP/±RTX	NA	better (s)<br>similiar<br>similiar	NA	NA
**Fritsch 2013 CM**	145	100% Larynx	OP/±RTX	NA	worse (S)	NA	NA
**Fritsch 2013 CM**	92	100% Oral cavity	OP/±RTX	NA	similiar	NA	NA
**Soriano 2008 CC**	62	50% Hypopharynx, 31% Oropharynx/ Oral cavity	OP/±RTX, neoadj. CTX	better (NS)	worse (s)	NA	NA
**Thariat 2008 CC**	51	85% Oropharynx	RCTX	Similiar	similiar	NA	NA
**Winzenburg 1998 CM**	26	NA	NA	NA	worse (NS)	NA	NA
**De Sampio 2004 CM**	17	100% Oral cavity	OP/±RCTX	Similiar	similiar ,if matched	NA	NA
**Yu 2008 CM**	15	66% Oral cavity,<br>27 % Sinonasal	OP/±RTX	Similiar	worse (ns)	NA	NA
**Erdamar 2000 CM**	10	100% Larynx	OP	NA	worse (ns)	NA	NA
**Luna 1990 CM**	9	65% Hypopharynx, 44% Oropharynx	OP/±RTX	Similiar	similiar	NA	NA
**Marioni 2008 CM**	9	100% Larynx	OP/±RTX	NA	similiar	NA	NA
**Coppola 1993 CM**	8	100% Oral cavity	OP/±RTX, pall. CT	worse (NS)	worse (ns)	NA	NA
**Begum 2008 CS**	53	40% Oropharynx,<br>26% Hypopharynx	OP/±RCTX	NA	NA	76% HPV(+)-Oropharynx, 6% HPV(+)-NonOro,	HPV(+)-BSCC better outcome (s)
**Ereno 2008 CS**	40	55% Larynx/ Hypopharynx	OP/±RCTX	NA	similiar	NA	NA
**Banks 1992 CS**	40	50% Oropharynx,<br>20% Hypopharynx	OP/±RCTX	NA	similiar	NA	NA
**Chernock 2009 CS**	28	43% Oropharynx, 57% Hypopharynx/ Larynx	OP/±RCTX	NA	NA	75% HPV-Oropharynx, 0% HPV-Nonoropharynx,	HPV(+)- BSCC better OS (s)
**Paulino 2000 CS**	20	25% Oropharynx,<br>25 % Larynx	OP/±RCTX	NA	similiar	NA	NA
**Choussy 2011 CS**	18	38% Hypopharynx 38% Larynx	OP/±RCTX	NA	worse (NS)	NA	NA
**Friedrich 2010 CS**	17	17% Oropharyx,<br>17% Oral cavity	NA	NA	NA	65% HPV(+)	NA
**Ferlito 1997 CS**	15	60% Larynx,<br>40% Hypopharynx	OP/±RCTX	NA	worse (NS)	NA	NA
**Larner 1993 CS**	15	38% Oropharynx,<br>13% Larynx	OP/±RTX	NA	worse (NS)	NA	NA
**Wieneke 1999 CS**	14	100% Nasal Cavity/Sinonasal	OP/±RCTX	NA	worse (NS)	NA	NA
**Klijanienko 1993 CS**	12	33% Hypopharynx, 33% Oropharynx	OP/±RCTX	NA	NA	NA	NA

Supplement 2: Literature review for treatment and survival data. CO = comparative study; CC =case-control study; CS=case series; OP=surgery, R(C)TX= radio(chemo)therapy, (2) NA=not assessed, (s)=significant, (ns)=not significant
